# Goals and Action Plans Across Time and Place—A Qualitative Study Exploring the Importance of “Context” in Person-Centered Rehabilitation

**DOI:** 10.3389/fresc.2022.788080

**Published:** 2022-03-14

**Authors:** Linda Eggen, Jette Thuesen

**Affiliations:** ^1^Danish Hospital for Rheumatic Diseases, Sønderborg, Denmark; ^2^Danish Knowledge Centre for Rehabilitation and Palliative Care, Nyborg, Denmark

**Keywords:** person-centered rehabilitation, rheumatology, everyday life, goal setting, action plans

## Abstract

**Background:**

Person-centeredness is increasingly addressed in relation to rehabilitation interventions. Collaborative goal setting and action plans are key measures in person-centered rehabilitation. There is a lack of knowledge about how person-centered goals and action plans developed away from the patient's everyday life are experienced by patients after discharge.

**Purpose:**

This aim of the study is to explore how patients with rheumatic diseases experience the relevance of goals and action plans after discharge from inpatient rehabilitation hospital stay.

**Methods:**

Individual narrative interviews were conducted with eight patients with rheumatic diseases, aged between 40 and 60. A convenience sampling strategy was applied. Data collection, analysis and interpretation of data were performed within a phenomenological-hermeneutic framework inspired by Paul Ricoeur's interpretative philosophy.

**Results:**

The analysis derived one core theme, “The relevance of goals and action plans is contextual” and three subthemes: “Admission—a protected bubble,” “Back home—a harsh reality,” and “Need for post-discharge support.”

**Conclusion:**

This study indicates that the relevance of goals and action plans to patients with rheumatic diseases is context specific. On the basis of the study, it is suggested that the context should be considered in rehabilitation practice, including the social network of the patients. This is in order to support patients in rehabilitation interventions to manage everyday life with disease after discharge to their own homes. Moreover, the concept of context in person-centered rehabilitation should be reconsidered. The study also concludes that there is a need for further development and research in follow up programs, as it is not clear what may constitute an optimal design of follow up support.

## Introduction

Person-centeredness is a key principle and characteristic of rehabilitation (1–3). Person-centered rehabilitation is widely recommended (4). Yet, person-centered rehabilitation may remain more of a rhetorical notion than standard rehabilitation practice, and professionals often perceive their care as being more person-centered than do the patients (5, 6).

According to Leplege et al., person-centeredness in rehabilitation has various historical origins and this implies diverse understandings (1). Person-centeredness in rehabilitation may imply paying specific attention to the person's subjective experiences, emotions and personal history (1, 7, 8), and may entail involving the person and families in goal setting and decision-making, individualizing and tailoring interventions to respond to the individual's needs, and viewing the person as an expert regarding their own needs (1). Person-centered rehabilitation has also been associated with empowerment and autonomy and with the recognition of careers and family members as crucial actors in rehabilitation (5). Moreover, person-centered rehabilitation may sustain personhood and increase coping and motivation (8) and may have a positive effect on occupational performance and rehabilitation satisfaction (6). Jesus et al. have suggested that person-centered rehabilitation is a way of thinking about and providing rehabilitation services “with” the person (5). Their PCR-model articulates that person-centered rehabilitation is embedded in structures and practices across the person-professional dyad, at the micro-system level and in the macro-system in which rehabilitation is delivered (5).

In healthcare in general, the outcomes of person-centered care have been described by McCormack among other scholars. According to McCormack, person-centeredness is about seeing the patient from a holistic perspective, with a focus on involving the patient and the patient's own values in joint decision-making. McCormack describes in his model the outcomes of person-centered care such as good care experience, involvement in care, feeling of well-being, and existence of a healthful culture (9, 10).

In line with this, person-centered rehabilitation may be expected to empower the individual and provide meaningful outcomes. These outcomes transcend time and place. Therefore, it is relevant to explore the relevance of person-centered measures across time and place.

Collaborative goal setting (6, 11, 12) and action plans (11) have been mentioned as key measures in person-centered rehabilitation. There is an extensive literature on goals and goal setting in rheumatic rehabilitation (12–16) as well as in rehabilitation in general (11, 17). Collaborative goal setting may have positive effects on psychosocial outcomes (17). Moreover, goals and accompanying action plans are expected to facilitate adherence to treatment plans and support a healthy lifestyle after discharge (11, 12, 14, 15, 18–20). In clinical rheumatology research, there is an increasing focus on post discharge support and how to extend the duration of the individual rehabilitation process after discharge (14, 15, 18–20) and establish new routines (15).

In Denmark, rehabilitation for people with rheumatic diseases is offered partly at a specialized rheumatology hospital and partly in municipalities. At Rheuma Rehab [pseudonym], a hospital for patients with rheumatologic diseases, specialized multidisciplinary rehabilitation is offered during hospitalization (21, 22). At Rheuma Rehab, person-centered rehabilitation includes specific goals and action plans that are prepared in collaboration between patients, relatives and an interdisciplinary team, viewing the patients as being able to manage their illness in everyday life (21).

Thus, the aim of this study was to explore how patients experience the relevance of the goals and action plans, that were developed at the hospital as elements in person-centered rehabilitation, after being discharged from the hospital and having returned to their own homes.

## Materials and Methods

### Design

Because the study aimed to gain an understanding of participants' lived experiences, a narrative approach was chosen. Data were collected through narrative interviews (23) and the analysis was inspired by the French philosopher Paul Ricoeur (24). Ricoeur's phenomenological-hermeneutic approach is a philosophical way of thinking, where a phenomenological understanding is combined with a hermeneutic interpretation and explanation of narratives (24, 25).

### Setting and Participants

The study took place at Rheuma Rehab Hospital where the participants were recruited at discharge by the first author. The study included patients with rheumatic diseases, who had completed a 10-day rehabilitation programme. The interview took place between three to five weeks after the discharge. A convenience sampling strategy was applied (26), and interview persons were recruited among the patients that were closest at hand. Given the exploratory nature of the study, we strived for variety in the participants' rheumatic diagnoses, ages and genders (26). Patients were excluded if they suffered from dementia or had other cognitive impairments. Ten patients indicated an interest in participation in the study. Two resigned before being interviewed, for personal reasons. The characteristics of the eight participants are shown in Table 1.

**Table 1:** Participants.

**Patient**	**Sex**	**Age**	**Diagnosis**	**Discharge/interview**
#1	F	>60	RA	5 weeks
#2	M	>60	RA	4 weeks
#3	F	>40	SpA	3 weeks
#4	F	>50	RA	3 weeks
#5	F	>50	RA	5 weeks
#6	F	>40	SpA	5 weeks
#7	F	>50	RA	3 weeks
#8	F	>40	RA	5 weeks

*RA, rheumatoid arthritis; SpA, spondyloarthritis*.

### Data Collection and Processing

Data were collected through telephone interviews due to the COVID-19 pandemic. An interview guide was developed in accordance with the aim of the study and with inspiration by Marianne Horsdal's narrative approach (23). The purpose of using narrative interviews in this study was to get the patients' narrative about the process of person-centered rehabilitation.

The narrative took it's beginning around the goal setting and action plan meeting—and proceeded to talk about the everyday life after discharge. Questions focused on specific events including the goal setting meeting. Moreover, questions were about how goals and action plans made sense after discharge.

Interviews were guided by an interview guide (Table 2) covering three phases and including initiating, exploratory and help questions in relation to “the goal setting meeting,” “the in-patient stay,” “the time after discharge” and “the future.”

**Table 2:** Interview guide.

Step 1	The aim of the study was presented to the participants
Step 2	The main phase started with an initial question: *If you think back at the rehabilitation stay, can you mention some particularly important events?* Exploratory questions were used to get more rich data on the events, e.g.: • Can you tell me more? • What happened? • How did that happen? • Who was involved?
Step 3	The next phase was about the processes and participants' use of goals and action plans within the hospital stay and after discharge: • When you think back, do you remember the meeting with the interdisciplinary team where the goals an action plans were developed? • Did you achieve your goals? What happened, can you tell a bit more about it? • What about the action plans, did it go as planned? What happened, can you tell more about it? • Now you are back home. When I mention everyday life after the discharge, what do you think about it, and can you describe how it is? • If you think back to the in-patient stay and until now, can you try to describe whether the goal and actions plans make sense to you now and whether they allow you to manage your illness in everyday life? • If you think about the future—how do you think the rehabilitation will affect your everyday life in the future (something you want to highlight in this regard?)

All interviews were audio-recorded and transcribed verbatim by the first author.

### Analysis

The analysis was performed in accordance with Ricoeur's phenomenological-hermeneutic approach to narratives. Ricoeurs approach is considered appropriate for a study whose aim was to create meaning and significance across data (24, 25). The analysis was performed by the first author of this article (LE).

The analysis entailed a dialectical movement between the whole and the parts of the content, in a hermeneutic spiral. The analysis process involved three steps, the naïve analysis, the structural analysis and the critical analysis and discussions.

1. In the naive analysis the text material from the interviews were read in order to deduce general understanding of the empirical data—that is, what the text was about. In the analysis, it appeared that the participants were predominantly talking about the in-patient stay in positive terms while the everyday context after discharge was considered challenging when they should work further with goals and action plans developed at the hospital.

2. In the structural analysis, the material was re-read and the structure of the text was uncovered in dialectical moves between explanation and understanding of what was said and what was talked about. A core theme and three sub-themes emerged. Within the narratives, there was a polarized talk about the hospital context and the everyday context. Moreover, the goals and action plans that was developed at the hospital seemed less relevant in the everyday context—or they were challenged by other tasks. The informants described the institutional context positively, as a protected bubble, opposite to the everyday context after discharge, which was described as a harsh reality, making it difficult to adhere to goals and action plans. To do so, informants needed after discharge support from the Rheuma Rehab Hospital.

3. In the critical analysis and discussion, the core theme and three sub-themes were discussed. The discussion expands the dialectic between explanation and understanding, including literature on contextual aspects in goal setting, action plans and person-centered rehabilitation.

### Ethical Statement

The study complied with ethical principles and with the practices of the Danish National Committee on Health Research Ethics (27), and with the guidelines from the Danish Data Protection Agency (28). In Denmark, this kind of research does not require approval from an ethics committee. Data use and protection was legally based on informed consent from participants, following General Data Protection Regulation, article 6, litra a. (29).

All participants were informed about the study and gave their oral and written consent to participate in the study prior to participation. They also gave consent to using the data for publication in a scientific journal. Participation was voluntary and anonymous, and the participants were informed about the opportunity to withdraw from the study.

## Results

The individual narrative interviews were conducted in March and April 2021. Eight participants with a rheumatic disease were interviewed 3–5 weeks after discharge from a rehabilitation in-patient stay at Rheuma Rehab. The telephone interviews lasted between 20 and 55 min. During the analysis, a core theme and three sub-themes emerged. The core theme: “The relevance of goals and action plans is contextual,” related to the fact that goals and action plans may be perceived as relevant during the hospital stay but that other goals and tasks may be more urgent after discharge. The three sub-themes that emerged were: “Admission—a protected bubble,” “Back home—a harsh reality” and “Need for post-discharge support.” The sub-themes illustrate the participants' experiences of the significance of the context for applying goals and action plans after discharge from a person-centered rehabilitation course.

### The Relevance of Goals and Action Plans Is Contextual

In the interviews, the participants describe the person-centered rehabilitation process as separated from everyday life. The stories reflect that goals and action plans, which may be perceived as relevant during a rehabilitation in-patient stay may be overshadowed by other tasks and challenges when you are back home.

### Admission—A Protected Bubble

The participants talk about being very excited and positive about the meeting in which goals and action plans were developed. One participant express being impressed with the resources allocated to the talk. Participants express that during the collaborative goal setting talk they felt seen, heard and listened to. The meeting unfolded as a collaborative process with committed and interested doctors, nurses, physiotherapists and occupational therapists who tried to understand exactly what the participants said, their challenges and what their needs were. The participants express that they felt involved in the preparation of goals and action plans. Goals and action plans involved implementing exercising and being active in everyday life, and being able to understand and manage one's illness.

The participants express that during the admission there was time and peace to do exercise. They talk about the Rheuma Rehab Hospital as a place where they were relieved from everyday duties and could focus on dealing with their illness. A female participant describes the in-patient stay as a “protected bubble,” a place where one is away from everyday duties.

“*When you are at the Rheuma Rehab Hospital you are in a kind of ‘protected bubble' and you do not have to cook, you do not have to work and you have time to rest and to exercise.” # 4*

During the admission, the participants received support from the interdisciplinary team in doing exercise as well as in conversations. As one male participant says, it helped with the motivation that they got support and that there were firm plans for what they were going to do:

“*When you are hospitalized, you are kind of served the motivation, and you are not allowed to have too many bad excuses, because there is always this day program.” # 2*

Participants describe that the conversations and seminars gave them an awareness and understanding of their illness. One woman says that it was at a nurse-led pain seminar that she kind of understood what it was all about, and that this was where she began to understand that the disease would not go away, but that she had to accept it. It helped to be able to deal with fatigue and pain. Both the exercise programs and conversations gave participants a recognition of having a chronic illness. Recognition and acceptance of the disease contributed to the participants being more open about their disease to the environment. Several of the participants expressed that at the time of discharge they were motivated and ready to transfer what they had learned from the hospitalization to everyday life.

### Back Home—A Harsh Reality

All participants describe that after the discharge, they experienced some challenges when trying to work further with goals and action plans in their everyday life. It is especially challenging to incorporate new habits, and as one of the participants who is still at the labor market describes, it is tough to get home to everyday life, which she was not prepared for at all.

“*When you come home, there is the harsh reality, then you have to go to work, and there are just many other things. You are not quite aware of what awaits you when you jump in at home, and then it becomes difficult” # 4*

Another participant describes that some days it feels like taking two steps forward and a half step back. A female participant talks about her challenges with her colleagues, in a way she has not done before. Many of the participants experience an everyday life with work and family where it can be difficult to prioritize exercising. Participants experience some days overshadowed by pain and fatigue. For some, it may be difficult to accept that they cannot do the same as before they became ill. One of the participants tells she is completely exhausted when she comes home from work and that she is so tired that she falls asleep when she sits on the couch. Additionally, another has acknowledged she can no longer keep working. Even though the participants know that exercising may benefit their physical and mental health, they do not always do enough exercising. The participants experience that the everyday life differs from the hospital context. The time and peace they had during the hospitalization is absent in their everyday life, and it can therefore be challenging to maintain habits and work with goals and action plans, when you do not have the support at home that you received during the in-patient stay.

### Need for Post-discharge Support

Several of the participants call for support or follow-up to work further with goals and action plans after discharge. The participants feel alone in everyday life, where most also have work, family, exercising and the disease to take into account. Several of the participants feel that they need help and support after discharge to be able to implement and maintain the new habits. A male participant who is very active in everyday life and true to his goal, describes coming home as a big change.

“*It is of course a tremendous challenge when you get home, because there is basically only one person who has to fill all those roles that you have now been confronted with when you were admitted.” # 2*

Participants are aware that they will have to live with their rheumatic disease for the rest of their lives, that they themselves have a responsibility to continue working on goals after discharge, both on good and bad days. Yet they find it difficult to accept that there are things they can no longer do and that they must ask for help. Asking for help can be a challenge because you want to fend for yourself, even if you had set a goal to become better at asking for help. A woman describes this:

“*I promised myself to ask for help, but it's hard to keep asking for help now after the discharge” # 6*.

On challenging days, participants miss having someone to talk to. Several participants suggest that relatives might be informed and involved in discussing goals and action plans at discharge, so that they could be supportive during periods that are challenging. While other participants suggest that it might be motivating and supportive that a nurse from the hospital would call after one, two or three months and ask how one is feeling, as a supplement to the admission.

## Discussion

The results of this study show that the participants were positive about the rehabilitation hospital stay. They felt relieved from everyday duties, they got support to prepare and follow rehabilitation goals, and they came to new understandings of living with a rheumatic disease. The results also show that the participants found it challenging to transfer goals and action plans to everyday life after discharge. While being at the hospital, the admission seemed far away from the participants' everyday lives. After the discharge, several participants needed help and support to deal with everyday life with the disease. Following Wade, rehabilitation is not only about what happens in the patient-professional encounter but also what happens outside the meeting (30). In the discussion, the importance of context and follow up programs are discussed.

The importance of the context in rehabilitation and considering the rehabilitation process as transcending time and place has been studied by several researchers. As already mentioned, there is an increasing focus on what happens after discharge from an inpatient rehabilitation stay: The need for post discharge coordination to create coherence and transfer learning to patients' everyday lives was documented by Stauner et al. in patients with rheumatic diseases (31). The need for post discharge support is further emphasized by this study, showing how the patients find it challenging to maintain habits and work with goals and action plans. In a qualitative study from Norway concerning the pursuit of goals after discharge, Hamnes et al. (15) showed that patients with rheumatic diseases experienced challenges in achieving goals after discharge, despite the fact that they had learned to use self-management strategies, also supported by this study. Moreover, the study by Hamnes et al. (15) showed how goals and action plans may change over time and how achieving goals may be challenged by external factors such as waiting lists for physiotherapy. The need for post discharge follow-up programmes in rheumatological rehabilitation is further documented by Dager et al. (12), Berdal et al. (18, 19) and Berdal (20). Dager et al. (12) found that structured goal setting and follow-up telephone calls enhanced motivation and may contribute to prolonged goal attainment. This is further supported by this study, where patients suggest a follow up call after admission. Berdal et al. (18) and Berdal (20) found that supportive follow up may improve self-reported physical function in patients with rheumatic diseases. Yet, they state that knowledge is scarce concerning what may constitute an optimal design of follow-up support (20).

The qualitative studies mentioned above, and the results of the current study show that context has a central significance in rehabilitation, which gives rise to discussing “context” in person-centered care and rehabilitation. McCormack's interdisciplinary theoretical framework for person-centered care and Jesus's person-centered rehabilitation model will be used below to discuss the results of our current study (5, 9, 10, 32).

In his interdisciplinary person-centered care framework, McCormack addresses the context with reference to the nursing and care environment in clinical practice. Person-centered care is a framework for rehabilitation that involves and strengthens the patient's goals in rehabilitation (8, 9, 21). Jesus addresses the context of his person-centered rehabilitation model in clinical practice (institution or rehabilitation center) and outside of clinical practice in the patient's own home. The importance of paying attention to the home environment is stressed by this study. Jesus indicates that it is essential that the person-centered rehabilitation is planned based on the context in which the patient experiences challenges with the disease. Jesus urges that person-centered rehabilitation in clinical practice involves discharge programmes and includes planning the return to everyday life (4, 5), so that the patient does not feel left behind after discharge. Wade supports this, stating that rehabilitation should be adapted to the context in which patients find themselves, based on a holistic biopsychosocial model of disease. According to Wade, rehabilitation should be based on an action plan and organized and structured according to the patient's everyday life (30, 33, 34). Yun and Choi (6) note that in person-centered rehabilitation, recognizing the patients/clients as whole persons should imply understanding them in a broad context. Also, Dean describes the “context” of person-centered rehabilitation (2, 7). According to Dean, contextual aspects is important to fully understand the patient's situation. Dean uses the term “person in context,” a term that requires recognition that the patient's biographical history is essential in rehabilitation. Dean thus focuses on the context that precedes the rehabilitation. Compared to the findings of this study, these theoretical perspectives all seem to suggest a broad focus on the context of person-centered rehabilitation in practice. Context should not be considered as restricted to what happens in the rehabilitation setting but should include the patient's biographical history and the everyday life after discharge as context, including the social network of the patient. Several participants suggest that relatives should be involved in planning the discharge.

Theory and other research on the relevance of the contextual factors, in conjunction with the results of this study, indicate that context should be considered in person-centered rehabilitation thinking and practice. The context can usefully be understood as the patient's context, across time and place. At the same time, the results of the study, other qualitative studies and a person-centered rehabilitation approach, indicate that coordination across contexts is required, to ensure coherence in the rehabilitation process. Rehabilitation may be considered a process that also continues when the patients are discharged to their own homes (35).

The study may have some limitations. A sample size of eight persons may not have allowed for full saturation in such a heterogeneous sample (26). Hence, no extensive conclusions can be made. Yet, as the results correspond with other studies, the findings and conclusions of this study seems valid. Another limitation may be that there was no involvement of patients or other partners in the research process. Patient and public involvement might have been beneficial but was not performed due to the limited timeframe within writing the thesis. Involvement of a patient research partner would have given us the opportunity to adjust the written material based on the patient's perspective and ensured the relevance of the interview questions (31).

## Conclusion

In person-centered rehabilitation, collaborative goal setting and action plans are used to make rehabilitation more person-centered. This study indicates that the relevance of goals and action plans to patients with rheumatic diseases is context specific. On the basis of this small study, it is suggested that the context should be considered in rehabilitation practice, including the social network of the patients. This is in order to support patients in rehabilitation interventions to manage everyday life with disease after discharge to their own homes. Moreover, the concept of context in person-centered rehabilitation should be reconsidered.

### Clinical Implications

This study indicates the need for rehabilitation professionals to be attentive to the patients' everyday life after discharge, not only in terms of goals and plans transcending the hospital stay, but also in terms of the social support available after discharge.

This study also stresses the need for post discharge support. There is a need for further development and research in follow up programs, as it is not clear what may constitute an optimal design of follow up support.

## Data Availability Statement

The raw data supporting the conclusions of this article will be made available by the authors, without undue reservation.

## Ethics Statement

Ethical approval was not provided for this study on human participants because the discussion piece is based on a student's thesis. The patients/participants provided their written informed consent to participate in this study.

## Author Contributions

LE has collected the data and completed the first draft of the manuscript. JT completed the first draft of the introduction. All authors have equally contributed to finish the manuscript. All authors contributed to the article and approved the submitted version.

## Funding

The APC is kindly funded by the Professional Danish Society of Rheumatic Nursing and Prof. Jette Primdahl, Danish Hospital for Rheumatic Diseases.

## Conflict of Interest

The authors declare that the research was conducted in the absence of any commercial or financial relationships that could be construed as a potential conflict of interest.

## Publisher's Note

All claims expressed in this article are solely those of the authors and do not necessarily represent those of their affiliated organizations, or those of the publisher, the editors and the reviewers. Any product that may be evaluated in this article, or claim that may be made by its manufacturer, is not guaranteed or endorsed by the publisher.
